# Substance Use Stigma and Community Drug Checking: A Qualitative Study Examining Barriers and Possible Responses

**DOI:** 10.3390/ijerph192315978

**Published:** 2022-11-30

**Authors:** Samantha Davis, Bruce Wallace, Thea Van Roode, Dennis Hore

**Affiliations:** 1 Canadian Institute for Substance Use Research, University of Victoria, P.O. Box 1700 STN CSC, Victoria, BC V8W 2Y2, Canada; 2 School of Social Work, University of Victoria, P.O. Box 1700 STN CSC, Victoria, BC V8W 2Y2, Canada; 3 Department of Chemistry, University of Victoria, P.O. Box 1700 STN CSC, Victoria, BC V8W 2Y2, Canada; 4 Department of Computer Science, University of Victoria, P.O. Box 1700 STN CSC, Victoria, BC V8W 2Y2, Canada

**Keywords:** substance use stigma, drug checking, substance use, harm reduction, overdose, fentanyl

## Abstract

Background: Community drug checking is an emerging response to the overdose crisis. However, stigma has been identified as a potential barrier to service use that requires investigation. Methods: A qualitative study explored how best to implement drug checking services to the wider population including those at risk of overdose. A secondary analysis of 26 interviews with potential service users examine how stigma may be a barrier to service use and strategies to address this. A Substance Use Stigma Framework was developed to guide analysis. Results: Drug checking is operating in a context of structural stigma produced by criminalization. People fear criminal repercussions, anticipate stigma when accessing services, and internalize stigma resulting in shame and avoidance of services. A perceived hierarchy of substance use creates stigma results in stigma between service users and avoidance of sites associated with certain drugs. Participants frequently recommended drug checking to be located in more public spaces that still maintain privacy. Conclusions: Criminalization and societal views on substance use can deter service use. Strategies to mitigate stigma include employment of people with lived and living experience from diverse backgrounds; public yet private locations that preserve anonymity; and normalization of drug checking while decriminalization could address the root causes of stigma.

## 1. Introduction

The illicit drug overdose crisis is an ongoing epidemic that continues to take lives at unprecedented rates. British Columbia, Canada has been identified as the epicenter in Canada, where approximately seven deaths per day are linked to unregulated substances most often including fentanyl [1]. Increasingly drug checking is being pursued as a potential response to the rapid emergence of synthetic opioids including fentanyl and the high rates of overdose related to the unpredictably of the unregulated drug market [2,3,4,5]. In Victoria, British Columbia, community drug checking sites have been implemented as a public health response to the ongoing overdose crisis and the unregulated illicit drug market through a community-based research project called the Vancouver Island Drug Checking Project [6]. In addition to providing anonymous, confidential, and non-judgmental drug checking services with rapid results, the project has conducted qualitative research aimed to better understand drug checking as a potential harm reduction response to the illicit drug overdose crisis and the unregulated illicit drug market [7,8]. The goal of the research was to hear about people’s experiences with drug checking, including people who use drugs, their family, friends, peers, and/or people who make or distribute drugs [7,8]. The central intention of the interviews was to gain a well-informed understanding of how people think about drug checking from a range of social locations, with the goal of making drug checking widely accessible, safe, and effective for all. Stigma related to substance use emerged as a dominant theme in our research that operated as a critical barrier that needs to be better understood in order to inform future implementation.

There is overall recognition of the need to address substance use stigma (SUS) as integral to responses to the illicit drug overdose crisis [9,10,11]. SUS is generally defined as the stigmatization of people who use drugs (PWUD) for using illicit substances [12]. In his early and well-known work on stigma, Goffman [13] identified stigma as a relationship where stigmatized qualities or stereotypes are applied to some by others, resulting in the stigmatized person being viewed as “tainted” or “discounted”. Understandings of stigma have shifted from Goffman’s interpretations of stigma as static to thinking of stigma as an ever evolving social phenomenon [14]. According to Tsai et al. [9], stigma is defined as a “process wherein people with a particular social identity are labeled, stereotyped, and devalued, unfolding within the context of unequal and often pre-existing power-relations, leading to discriminatory behaviour against people with the stigmatized identity”. According to Tyler [15], the most common form of violence in democratic societies is stigma despite rarely being considered a form of violence or abuse of power. 

Moreover, existing literature has identified that attention is often focused on stigma at the individual level, which overlooks the realities of social, political and cultural contexts [16,17]. However, stigma can operate at different levels throughout society and function as a tool to control and oppress [18]. This has been referred to as “stigma-power” and is typically theorized as the stigmatizer having motivations to maintain status, wealth, and power, enforce social norms, and marginalize those viewed as unfit for society, and as such employs stigma processes that are easily, effectively, and indirectly accepted and enacted in society [18]. Based on this interpretation of stigma-power, the stigmatizer is not necessarily limited to a powerful individual or group, but also permeates the general public and the individual, therefore it can exist at the individual, interpersonal, and structural level. While stigma-power exists at each level, it is more easily identified at the structural level. This article looks at systemic and criminalization stigma as examples of structural stigma as the structures of agencies and laws that interact with substance use exemplify how stigma-power is at play. For example, systemic stigma is enacted and enforced by agencies, institutions, and influential individuals within groups of people and targets those who are stigmatized by attempting to manage risk and govern their interactions [19]. An important factor of systemic stigma is it protects the stigmatizer from repercussions of discrimination, because the stigmatization is embedded in norms, policies, or resources and the goals of stigmatization are achieved at the macro level Tsai et al. and Link and Phelan [9,18]. Additionally, criminalization is a form of systemic stigma that is closely related to substance use and is a common and effective way for governments to “control and exclude persons who are defined as threatening to an existing social order” [20]. It is often enacted through street policing and can result in reduced access to harm reduction services, rushed injection, increase in disease transmission, and increased risk of overdose [21]. Criminalization can be more common for people who use substances and who are experiencing poverty and/or are racialized or experiencing other intersecting identities [20]. It is an effective tool to “other” PWUD and maintain existing hierarchies in society and is considered a way to shift public attention from systemic inequities [20].

This paper seeks to broaden the existing conceptualizations of stigma in current literature to allow for consideration of how difference and power are created and maintained, as well as tangible ways to challenge existing norms, cultures, and structures that sustain SUS. As discussed by Parker and Aggleton [14], stigma and stigmatization function “at the point of intersection between culture, power, and difference”. Therefore, it is important to understand how people may be experiencing these different levels of stigma, as well as the interactions between them to better understand how they may be influencing successful implementation of drug checking services. This is achieved through the perspectives of the participants in this study. 

Substance use stigma prevents people from engaging in harm reduction practices [8,9,22]. Further, Corrigan and Nieweglowski and Tsai et al. identify stigma as a main hindrance in adequately responding to the opioid crisis through harm reduction practices [9,22]. There are many ways of thinking of and conceptualizing both stigma and substance use stigma in the existing literature. The substance use stigma framework from which this article will draw includes some of the most common definitions of stigma in the existing literature. 

Tsai et al. [9] identify 6 types of stigma: structural, public, enacted, courtesy, internalized, and anticipated. Additionally, Corrigan and Nieweglowski [22] identify three types of stigma: label avoidance, public stigma, and self-stigma. Earnshaw and Chaudoir [23] identify internalized, anticipated, and enacted stigma. Other scholars discuss structural stigma, which often includes discussions around laws and regulations [24]. There is a general theme of substance use stigma existing at three levels: an individual level where stigma exists within individuals, an interpersonal level where stigma exists within interactions between people, and a structural level where stigma exists at a policy level. 

As drug checking is increasingly being implemented as a harm reduction response to overdose there is a need to examine how stigma may uniquely be enacted and responded to in these newer services. In its simplest form, drug checking is the process in which drugs are tested for components that are not expected in a supply and/or that could cause overdose or otherwise undesirable reactions [25,26,27,28]. Drug checking is a harm reduction response to help prevent PWUD from consuming substances they did not intend to purchase and to provide the opportunity to make informed decisions about drug use [8,29]. This approach has been adopted across European countries and in some cities in North America, which accept illicit drug use will continue despite prohibitions and opt to offer special services to drug users [25,30].

Dancesafe, an American drug checking organization, was founded in 1998 in response to the risks posed by adulterants (harmful additives) being found in ecstasy (Dancesafe.org, accessed on 31 January 2022). This response to party drug related overdoses was largely mirrored across Europe and America [31]. Dancesafe takes the stance that they neither condemn nor condone drug use, rather they provide non-judgmental support to PWUD. This shows that the drug checking movement began in response to young people who used party drugs, such as Ecstasy, in recreational setting likes raves and who were not being reached by existing prevention programs and supports for PWUD [25].

We have reached the sixth year of the publicly declared overdose crisis in British Columbia and there is no sign of improvements [32]. Bardwell and Kerr and Kerr and Tupper suggested drug checking may be an effective response to the opioid overdose epidemic [33,34]. While Canada may be considered progressive in its regulation of cannabis, Larnder and Burek [32] and Wallace et al. [8] state it is the failed drug policies that are eresponsible for thousands of overdose related deaths. The literature illustrates that drug policy is not likely to change as quickly and effectively as is necessary for lives to be saved, thus highlighting the need for drug checking as a means of harm reduction in the meantime. Existing research has not found that the presence of drug checking sites has resulted in an increase in PWUD, nor has it found that individuals who use drug checking services use more drugs than those who do not [25]. This article contributes to the existing literature by providing insight and recommendations to reduce stigma related to drug checking from community members who are living through the overdose crisis. 

We aimed to specifically explore the role of SUS as it relates to community drug checking from the perspectives of people who use and/or sell drugs and others impacted by the illicit drug market. A secondary analysis of qualitative interviews was conducted to explore in depth how stigma was being experienced and could be operating as a barrier within drug checking services, and potential strategies that could address this. We conducted this research with a critical harm reduction and social justice approach that seeks to transcend neoliberal perspectives of harm reduction [7,35,36,37], and developed a multilevel analytic framework to guide this research that combines critical perspectives on stigma and how they can operate and intersect specific to a drug checking context [9,18,19,20,21,23]. We used this resulting Substance Use Stigma Framework to better understand the experiences of SUS within drug checking services and potential ways to navigate resulting tensions.

## 2. Materials and Methods

This qualitative study was part of a community-based research project in Victoria, BC, Canada that implements and operates community drug checking sites as a harm reduction approach to the illicit drug overdose crisis and the unregulated illicit drug market. Ethical approval was provided from the Human Research Ethics Board at the Island Health Authority (J2018–069). This research project was a collaborative inquiry with both university (B.W., T.V.R. and D.H.) and community researchers with local harm reduction organizations. The intention was to include individuals with academic training and skills and those with established trusted relationships with potential participants. 

### 2.1. Sampling 

The study sought the perspective of people who use and/or sell substances, and others impacted by the illicit drug market, such as family and friends who might benefit from drug checking services. An earlier study [8] primarily reached individuals who utilize inner-city resources, like harm reduction and health services. The objective of this study was to both include and expand beyond this demographic to reach individuals who are less likely to utilize inner-city resources, as well as people who make or distribute substances. Everyone who expressed interest was interviewed. Handbills, posters, and emails to local services were utilized to recruit participants because it allowed for third-party recruitment and increased potential to reach a wider audience. Because the objective was to reach beyond inner-city service users and those who already access harm reduction sites, recruitment posters and emails were sent to services and sites that were not explicitly focused on substance use nor inner-city health and homelessness such as neighbourhood houses, community health centres, food and employment programs, etc. Word-of-mouth also functioned as a significant recruitment strategy and no one who expressed interest was refused an interview. In the end, while most participants were not accessing existing harm reduction services, five participants had accessed drug checking. Participants were provided with a CDN$20 honorarium. 

A total of twenty-six semi-structured interviews were conducted, eleven of which were conducted by the lead researcher, eight by interviewers with the drug user organization, and seven by the partnering harm reduction organization. Interviews typically lasted between 15 minutes to just over an hour, with an average time of 30 minutes. We also asked demographic questions as well as questions related to substance use and overdose, and use of harm reduction services. Most questions focused on how drug checking could best be implemented, for example; what would you hope for in a drug checking service, if you could design a perfect drug checking service how would it operate, how would a service fail to meet your expectations and what barriers do you face in accessing drug checking. Recorded interviews were transcribed verbatim by graduate research assistants and coded using NVivo 11 (led by T.V.R.). 

### 2.2. Data Analysis

This secondary analysis focused on exploring SUS, particularly aiming to understand how it creates barriers to accessing drug checking services and potential strategies to address these. We reviewed existing literature considering how stigma is currently discussed and theorized across disciplines and within the substance use and drug checking context. As no consistent framework from which SUS is typically analyzed exists, we developed a Substance Use Stigma Framework based on existing literature which we utilized as an analytical tool.

For this SUS Framework, we drew heavily on Tsai et al.’s [9] typology of stigma related to substance use which highlights how stigma influences several facets of our lives, as well as other broader conceptualizations of stigma recognizing that stigma may operate at different levels and intersect across levels and with systems and services resulting in experiences of structural violence, and reinforcing power and oppression [14,15,18]. Our analytical framework considers SUS at three levels; individual, interpersonal, and structural. Within these levels we considered two types of SUS at each level. At the individual level, we looked at anticipated stigma and internalized stigma; at the interpersonal level, we looked at enacted stigma and episodic stigma; and at the structural level we looked at criminalization stigma and systemic stigma. While the framework presents each level of stigma as distinct levels, we recognize the complexity of SUS and the significant intersections of each level. For these reasons, the framework intentionally avoids a hierarchical approach as a strategy to acknowledge the creation and maintenance of SUS at each level, but also highlights that each level is not mutually exclusive and cannot exist without the others. A description of the framework and associated definitions and supporting references are given in Table 1.

Data analysis began with SD and BW reading the transcribed interviews. A preliminary coding framework was then developed using the SUS Framework’s six types of stigma function as the parent nodes and associated child nodes within these to capture “stigma barriers” and “possible solutions”. Initial coding was conducted with SD and BW coding two transcripts separately to ascertain framework fit and coding reliability. The transcripts were then deductively coded to this framework in NVivo 11. Findings were then grouped to identify experiences of stigma at each level, how these can operate as barriers to use of drug checking, and potential strategies to address these. 

## 3. Results

There were 26 participants of whom 17 identified as female, 8 as male, and one participant choosing not to disclose (Table 2). There were six who identified as Indigenous and 10 who identified as lesbian, gay, two-spirit, queer, bisexual or another sexual orientation. Participants predominantly resided in urban municipalities, in stable housing, and were wage earners and/or on disability benefits. The majority [22] reported they regularly consumed illicit drugs, with about half of these reporting daily use [11], about half (n = 10) reporting they usually used alone, and the majority [20] reporting that one of the common locations for consumption was their own home. Participants included family members of people who use drugs, notably parents, some who also identify as using drugs themselves. Few participants reported using harm reduction services.

Here, we present findings according to the Substance Use Stigma Framework’s three levels with six domains. For each level, we explore experiences of SUS, potential barriers it may cause, and potential responses or solutions to these barriers. 

### 3.1. Individual Level: Anticipated and Internalized SUS

At the individual level, we heard that the experience of anticipated (the expectation one will experience prejudice, discrimination, or judgement because of substance use) and internalized (the process of believing in and internalizing negative feelings around substance use) SUS was pervasive. 

Participants noted they felt too embarrassed or afraid to access drug checking due to a fear of being seen and expected others to feel similarly. For example, as this participant stated: “stigma, others seeing you” was a significant barrier to access and another that they were “always conscious of who could see them”. For participants with intersecting identities, in particular sex work, the anticipation of additional stigmas was a serious barrier to access and would lead to avoidance of accessing drug checking services as this quote highlights:

“You know, as a sex worker, having been so deeply stigmatized and still stigmatized for that, I’m hypersensitive to any additional stigmas, so, yeah, downtown doesn’t really work for me in that regard”. 

Participants with higher paying jobs or more normative careers also identified anticipated stigma as a serious barrier because they did not want to be associated with substance use or with those appearing to be poor or experiencing homelessness. For example, one participant said: “Yeah, people don’t want to get seen by people like their customers, their clients, or co-workers” and further noted that “I think people who live in the suburbs and have real jobs are less likely to be OK with it. Or to be seen as being associated with it”. Another participant indicated that this fear can extend to personal relationships including family: “I’m sure there’s so many people out there using, that are working, or, and their family doesn’t know”.

Accessing downtown resources was described as intimidating and a barrier to drug checking if located and designed as an inner-city service because of this internalized or anticipated stigma. This included harm reduction services including safe injection sites or overdose prevention services as well as non-profits and drop-in centers. Further, we heard similar stigma barriers to accessing drug checking services in small towns or on reserve as there is little opportunity to remain anonymous. This quote illustrates the high levels of stigma people may face when accessing such services and attempts to separate from this:

“But even just walking in here, today, I was just like was layered with stigma and shame, and you know, all of that and I kind of had to say to the security guard, which was quite honest … to make him very aware that I’ve never been here before and I don’t belong here”.

People expressed feeling like they had to justify their substance use with a reason like trauma or self-deficiency, viewing themselves as “at risk” or “an addict” [P8] if they used a safe injection site, and feeling concerned about looking like those around them. In contrast, some participants expressed pride at not having internalized nor anticipated stigma as a person who uses drugs and accessing harm reduction services. One participant expressed how they have “been in that world for a long time” and are pretty open about substance use, but they recognized this was not the case for most other people who use drugs.

We also heard participants mention a hierarchy of substance use, where using opioids, for example, is viewed as “pretty bad”, but doing MDMA, “that’s fine”. This internalized SUS could prevent some people from accessing spaces that serve individuals who use those substances due to how it is perceived. Internalized stigma was also attributed to public health messaging around substance use, like “don’t use alone” which was described as not realistic and has just led people to a place where they do not want to tell people they are using alone. The suggestion we heard was to find ways to open up conversations around substance use as this participant discussed: 

“So, I’ve had so many conversations with people where like if you’re using alone how are we doing that safely? Who are you calling? Like what does that look like? Like, this is what I do when I use alone. Like having those conversations with people has been super important because I think people feel really ashamed to be like “Oh, I use alone”. 

Potential responses to address individual level SUS focused on service models and sites that address the fear of being seen accessing drug checking services, respectful staff, as well as promotional campaigns to get your drugs checked that take care to avoid stigmatizing messages. General public settings that are less stigmatizing were suggested such as pharmacies, medical clinics, community laboratories, grocery stores, gas stations, and recreation centres. Specifically, pharmacies were described as public sites but private enough to maintain a level of anonymity and suggested a private booth for the checking process. Another solution was services that do not require the physical presence of another person such as mail in or online services, drug checking kits in dispensers in public bathrooms, drop off services, and personal testing kits that can be disposed of after use. Others recommended more confidential service options such as outreach and mobile and mail-in services. 

### 3.2. Interpersonal Level: Episodic and Enacted Substance Use Stigma

Fewer participants identified specific examples of personal experiences with enacted (when others behave in a way that communicates judgement, prejudice, or disapproval) and episodic (isolated events where stigmatization occurs over time) stigma. It was more common for participants to identify what enacted and episodic stigma would look like to them, including judgement, rudeness, blaming, and a lack of professionalism or how they have witnessed others being stigmatized. For example, enacted stigma was described as “somebody being rude or being judged for being a junky”. There were also instances of enacted stigma being reproduced in the interviews, for example, some language that was used by participants to describe different groups of people was both stigmatizing and stereotyping as well as judgement towards others based on substance type, i.e., the hierarchy of substance use mentioned previously. 

In this study, some participants stated they had not experienced enacted or episodic stigma, especially when accessing community drug checking, where they noted they felt welcome and safe. While we heard that some people many may feel unsafe disclosing substance use to their family doctor, a nurse, or drop-in clinic doctor, participants identified drug checking sites as hopefully different. One participant indicated that “Anybody but The Man” should be running drug checking, such as:

“People that care; harm reduction workers, support workers, people that have compassion for the safety and viability of others but still have no opinion about drug use one way or another. People that accept, acceptance, people that accept drugs as part of their community.” 

As noted, a potential response to enacted and episodic stigma included hiring staff who are/have used substances or who are committed to harm reduction for people who use substances, viewing them as equally deserving of respect and kindness. Overall, participants felt people with lived experience would be more accepting of people who use drugs and that substance use is part of their community. However, we heard that the definition of and portrayal of a “peer” varied. Throughout the interviews, two levels of people who use drugs were typically identified consistent with a hierarchy of substance use. One level is those who use street drugs, more commonly thought of as heroin/opioids or methamphetamines. The other level being so-called recreational substances, like MDMA or cocaine. Some participants thought it was important to tailor drug checking sites to both groups of people using substances, for example having “hipster” drug checking in a storefront for the recreational group and then continuing to have drug checking in harm reduction spaces in urban or downtown areas. Another recurring theme was to reduce interpersonal stigma by normalizing drug checking, either by promoting it by word of mouth, advertising it like designated driver programs, and putting drug checking sites in common spaces “where normal people go all the time” or in spaces where people already feel safe as supported to also reduce individual stigma.

### 3.3. Structural Level: Criminalization and Systemic Substance Use Stigma

Structural stigma included mentions of stigma systemically produced and maintained in societal institutions and includes criminalization stigma, usually identified as fear of police and authorities relating to the illegality of certain substances. We heard the multiple barriers to accessing drug checking as it relates to police presence and criminalization and how this embeds structural stigma. 

Participants noted fear of being watched and targeted by police, and how drug checking is hard to access if avoiding or hiding from police. One participant stated: “police presence isn’t causing harm reduction. Police presence is causing death” and that drug checking needed to ensure police would not be on or outside of sites. We heard that substances being illegal makes drug checking also illegal, resulting in fear and avoidance of the service due to potential criminal repercussions. It was clear that any association with police would discourage accessing community drug checking and breakdown and prevent any opportunity for trust. In particular, those distributing drugs were noted as likely to avoid drug checking because of risk of criminalization and potential for police to gather information. A fear of an internet trail that could identify substance use or distribution (even when no personal information was provided, collected, or recorded) was also indicated. A number of participants identified a fear of getting caught jeopardizing their employment or custody of their children.

We also identified that systemic SUS was attributed to promotion in the media and general public of certain areas that stigmatized entire areas because of associated substance use. In particular this applied to areas with harm reduction sites, as well as systemic stigma attached to non-profits, which can discourage some people from accessing them. For example, this participant noted the systemic stigma present when harm reduction sites were initially implemented in BC: 

“People were like “oh my god, they’re going to be sitting there just shooting up”. Well, what do you think they are doing anyways, right? I just, it was just so shocking. I lived on [the mainland] at that time, and it was just, the news and everything is like “this horrible place where people are just sitting around shooting up”, it’s like what do you think they’re doing in the [area of city]? They’re not having a tea party, you know. 

Potential responses to help mitigate systemic stigma again included suggestions to normalize the existence of drug checking, potentially by simply creating more sites as a means to mainstream drug checking. Other options for normalizing drug checking included promoting it on billboards and other public campaigns around drug checking and positive drug culture so people can see themselves and legitimize the service. Professional storefronts were also suggested to legitimize drug checking. Further, we heard several participants call for decriminalization, the regulation of the drug supply, institutionalizing drug checking, developing more treatment centres, and shifting to treating addiction as a medical problem to address structural stigma. Participants also noted shifting systemic stigma through promotion of harm reduction in schools and an “invitation to drug checking through education”, shifting messaging to younger people from abstinence to safe use including drug checking as it is a more realistic approach. 

## 4. Discussion

This study explores how substance use stigma may impact community drug checking from the perspective of potential service users. In our research on how to implement drug checking as a response to the overdose crisis we consistently heard how stigma was a potential barriers and how drug checking needed to address stigma to be effective. We drew from existing literature on substance use stigma to take a unique look at how stigma may impact the introduction of drug checking as a harm reduction response to overdose. By examining substance use stigma at the individual, interpersonal and structural levels and how community drug checking projects can navigate these barriers and mitigate their impacts we have applied existing stigma theories to inform drug checking as a public health intervention. Overall, while all sources of stigma presented barriers, participants described the risk of criminalization and the anticipation of being poorly treated appear to be the most significant barriers related to stigma and that deter service use.

Being criminalized is clearly stigmatizing and the presence of police erodes safety and trust for people who use drugs to access drug checking and other harm reduction services and sites services [8,21,38]. Furthermore, criminalization disproportionately impacts Indigenous, Black and other racialized groups, expectant birth-givers, transgender and gender diverse folks, sex workers, and others who face oppression in daily life [20,21,39].

Decriminalization is a structural intervention that has the greatest potential to reduce substance use stigma both by normalizing substance use, removing criminal repercussions, and removing the morality, the view that something is either right or wrong, from our perception of people accessing drug checking sites [40,41]. Drug checking and decriminalization are well-aligned and arguably instrumental to each other in the absence of widely available regulated drug supply [42].

A clear theme from this research was having access to drug checking in spaces that are not currently stigmatized was suggested to ease the anticipation of being stigmatized while accessing a drug checking site. Participants identified feelings of fear and embarrassment when accessing or considering accessing drug checking sites, especially where community drug checking is viewed as an inner-city service within a non-governmental organization serving a clientele assumed to use drugs. However, cultural safety for people who use drugs varies and accessibility for some may be inaccessibility for others [43]. Fear of association with these types of services may be rooted in structural stigma as well as a hierarchy of substances identified by some of the participants. For example, there was a clear distinction between substances typically viewed as “street drugs” versus “party drugs”. Others have identified this hierarchy of substance use, where drugs are considered more socially acceptable, including marijuana, alcohol, and ecstasy [44,45]. The implications of SUS in this form could result in shame for those using substances that are considered more harmful and the avoidance of drug checking services if the site does not seem safe or relevant for them. Enacted stigma is defined as engaging in behavioural manifestations, including discrimination and social distancing, based on views of social and cultural unacceptability [9,46]. It was made clear in the interviews that enacted stigma exists amongst people who use substances, with stigma towards others based on substance type, and is therefore not limited to service providers and people who do not use substances enacting SUS. It is possible the anticipation of stigma while accessing drug checking sites could be linked to this hierarchy of substance use as some identified the avoidance of accessing drug checking sites and non-governmental organizations as they did not feel they “belonged” in those spaces. 

Rather than location drug checking within inner-city harm reduction sites, participants frequently described drug checking as needing to be openly public yet still anonymous. General public and commonly accessed locations were perceived as less stigmatizing than harm reduction sites that help counter fear of identification when accessing services and potentially normalize drug checking. At the same time, privacy is essential and ensuring privacy within these public settings is necessary. While this may help to create accessible and appropriate services for some people, these spaces may be inaccessible and unsafe for others including racialized folks, women, transgender, non-binary, and gender diverse folks and people living in poverty [43]. Further, some medical professionals and spaces have been found to have negative attitudes towards people who use substances and often lack adequate training and education around substance use and critical harm reduction approaches [47]. Therefore, it is critical to recognize the experiences of interpersonal stigma that can occur within these environments and consider strategies to create safer environments. 

The employment of people with lived and living experience (PWLLE) as service providers was consistently identified as a strategy for facilitating trust and reducing the stigma that service users feel when services. PWLLE bring a unique perspective to harm reduction services and have a personal understanding of how to implement effective client-centred services, policies, and programs [38,48]. Employment of PWLLE is also an overarching strategy that is being recommended to help address structural determinants of inequities including stigma at the structural level [49,50]. 

Our prior work has indicated how critical employment of PWLLE is within harm reduction services to counter the poor treatment and stigma service users have experienced (8). However, we heard that the definition of who fit as a ‘peer’ varied as much as the respondents did. Participants were looking for peers that reflected their own social location and the substance types they use whether that be an opioid, stimulant or psychedelic. Including peers from a broad range of backgrounds and substance types can help reduce stigma associated with substance use and allow service users to be more comfortable and better identify services as appropriate to their needs [6]. Care needs to be taken that this does not inadvertently result in exclusion of those with the most structural disadvantage and further increase stigma. 

Turan et al. [51] discusses the rise of the term “intersectional stigma” which builds on Kimberley Crenshaw’s work on intersectionality [52]. Tsai et al. [9] discuss how each existing type of SUS intersect with one another and serve to reinforce the harms of each type, at each level. Thinking about the intersection of SUS with other factors in peoples lives allows for a holistic approach to understanding the issues SUS can cause for those impacted by it. This intersection of each type of SUS and the diverse identities of those who are stigmatized creates a complex and nuanced circumstance that inevitably reproduces more extreme cases of marginalization and oppression. For example, the impact that SUS has on a middle class, middle aged white male is significantly different than that of the impact SUS may have on a Black woman experiencing poverty or an Indigenous non-binary person living in a rural area. The interaction of SUS with privilege is an important nuance to understand and a particular focus needs to be given in further research to the role SUS has in reproducing other structural barriers. 

The normalization of drug checking was a recurring theme to addressing substance use stigma while the ongoing criminalization of drugs and people who use drugs hinders such efforts. Recommendations to normalize drug checking included health promotion campaigns similar to designated driver campaigns, implementing drug checking within common spaces where people already go or where people already feel safe, and promoting the positive benefits of drug checking more than the harms and risks of drug use. Unfortunately, public health promotion campaigns and even anti-stigma campaigns directed at substance use and people who use drugs too often reinforce SUS highlighting the critical importance of care with messaging to address stigma at multiple levels [53,54]. For example, we heard that while safer use messages such as “Don’t Use Alone” that aim to shift away from anti-drug messaging, can also convey blame and shame. It is important to note, however, that some evidence shows the effectiveness of negative campaign in reducing behaviours like tobacco use [55]. In this particular instance, the focus is on stigma-reduction related to illicit substance use and therefore policy change should focus on evidence relating to such data. 

## 5. Strengths and Limitations of the Study 

A strength of this study is that it seeks the perspectives of people who use substances to understand the impacts substance use stigma has on accessing community drug checking. This study was conducted by a research team that includes community partners from local harm reduction and drug user organizations in Victoria, BC, Canada. A comprehensive substance use stigma framework was developed that incorporates critical theoretical perspectives, appropriate to drug checking, to support analysis and understanding of the multiple and intersecting levels of stigma that can influence successful implementation. This research study was impacted by the COVID-19 pandemic, which resulted in the research team ending interviews earlier than initially intended. Overall, while we were successful in reaching a diverse group within Victoria, BC, who were not accessing harm reduction services, we were unsuccessful in recruiting men in the trades, who have been disproportionately affected by the overdose crisis, or youth. Further research needs to explore substance use stigma for people outside of urban centres, including people in rural or reserve communities, to determine how stigma may be operating within these contexts. Future research is also needed to understand the level of acceptability of community drug checking moving into new spaces, such as pharmacies, medical buildings, and more and what types of promotion and messaging would be most effective for addressing stigma at different levels.

## 6. Conclusions

Community drug checking is increasingly being viewed as one potential response to the illicit drug overdose crisis in which unpredictable and potent drugs are linked to unprecedented levels of overdose. However, potential service users identified numerous ways in which substance use stigma operates across levels to create barriers to accessing such services. The risk of criminalization and the anticipation of being poorly treated, both consequences of a wider systemic substance use stigma, were significant barriers that can deter use of services. Further, we identified a perceived hierarchy of substance use with greater stigma associated with certain types of substances, that resulted in stigma towards others and avoidance of sites and areas associated with such substance use. Structural interventions such as decriminalization are needed to address root causes of stigma at all levels. Strategies to mitigate these tensions within the current context of criminalization included employment of people with lived and living experience from a wide range of backgrounds; public yet private locations that preserve anonymity; and normalization of drug checking.

## Figures and Tables

**Table 1:** Analytical Framework.

	Level	Characteristics	Content
Substance Use Stigma	Individual Level	Internalized	According to Earnshaw and Chaudoir [23] internalized stigma refers to the process of believing in the negative feelings imposed on those who use substances, it is the internalization of negative beliefs and feelings associated with substance use. Tsai et al. [9] explain internalizing stigma can result in maladaptive behaviours, such as withdrawing from care or resources. This can lead to the belief that one’s status in society is less valuable than others [9].
		Anticipated	The expectation that one will experience prejudice, discrimination, or judgement is considered anticipated stigma, as defined by Earnshaw and Chaudoir [23]. It is common for an individual who anticipates stigmatization to adapt their behaviours out of fear of rejection by others and avoid services or means of care that could be beneficial to their well-being [9]. For example, one might avoid a community drug checking service, which could lead to negative impacts for that individual, such as consuming unknown or fatal substances.
	Interpersonal Level	Episodic	Episodic stigma refers to isolated events where stigmatization occurs over time, rather than on a consistent basis [19]. For example, if an individual experiences substance use stigma as they enter a safe consumption site or a community drug checking site at least once, but not constantly, they would be experiencing episodic stigma.
		Enacted	Enacted stigma is demonstrated when others behave in a way that communicates judgement, prejudice, or disapproval (and stigmatization) of a stigmatized individual through actions such as social distancing or avoidance [23]. This often takes place when the public perceives a stigmatized individual as dangerous or to have moral failings (often based on stereotypes) and discriminates against or avoids that individual for that reason [9]. Enacted stigma also means the individual who has been stigmatized believes they have been treated in a way that is discriminatory and/or prejudiced [9]. Enacted stigma becomes structural when the attitudes and beliefs are merged with cultural norms, laws, and policies.
	Structural Level	Systemic	Systemic stigma is enacted and enforced by agencies, institutions, and influential individuals within groups of people and targets those who are stigmatized by attempting to manage risk and govern their interactions [19]. The act of using stigma as a resource to oppress has been identified as stigma power [18]. Link and Phlan [18] describe the relationship between systemic stigma and stigma power as a means to oppress or maintain the oppression of stigmatized groups and to reinforce stigmatized identities in society. An important factor of systemic stigma is it protects the stigmatizer from repercussions of discrimination, because the stigmatization is embedded in norms, policies, or resources and the goals of stigmatization are achieved at the macro level [9] and Link and Phlan [18].
		Criminalization	Criminalization is a form of stigma that is closely related to substance use and is a common and effective way for governments to “control and exclude persons who are defined as threatening to an existing social order” [20]. Structural stigma is manifested in police responses to health needs such as problematic substance use and mental health [24] Criminalization can be more common for people who use substances and who are experiencing poverty and/or are racialized or experiencing other intersecting identities [20]. It is an effective tool to “other” PWUD and maintain existing hierarchies in society [20]. One example of criminalization stigma is policing around community resources like safe consumption sites, needle exchanges, or community drug checking, often leading to mistrust in a service and decreased access [21].

**Table 2:** Characteristics of the sample (N = 26).

Characteristic	Number (n)
**Gender**	
Male	8
Female	17
Other (gender queer)	1
**Age in years**	
20–24	1
25–29	2
30–44	12
45–60	10
>60	1
**Identify as Indigenous (First nations, Métis, Inuk (Inuit))**	
No	20
Yes	6
**Sexual orientation**	
Lesbian, gay or bisexual	8
Heterosexual or straight	16
Other	2
**Frequency of illicit substance use**	
Daily	11
Weekly	7
Occasionally, not every week	4
Never	4
**How often do you use alone?**	
Never	2
Sometimes	7
Usually	11
Always	1
Don’t know	0
Does not apply	3
**Where do you live?**	
Victoria	13
Esquimalt	4
View Royal	1
Saanich	4
Sooke	2
Langford	2

## Data Availability

Not applicable.
